# Why do parents enrol in a childhood obesity management program?: a qualitative study with parents of overweight and obese children

**DOI:** 10.1186/s12889-017-4085-2

**Published:** 2017-02-02

**Authors:** Kamila Davidson, Helen Vidgen

**Affiliations:** School of Exercise and Nutrition Sciences, Faculty of Health, Queensland University of Technology, Centre for Children’s Health Research, 62 Graham Street, South Brisbane, QLD Australia

**Keywords:** Child, Obesity, Overweight, Recruitment, Program

## Abstract

**Background:**

Despite the high prevalence of childhood overweight and obesity enrolment to weight management programs remains difficult, time consuming, costly and has limited effectiveness. The aim of this paper was to explore parents’ perspectives on factors that influence their decision to enrol in a program to address their child’s weight.

**Methods:**

Semi-structured qualitative telephone interviews were undertaken with 21 parents of primary school aged children above the healthy weight range who had enrolled in a healthy lifestyle program. Questions were developed and analysed using the Theory of Planned Behaviour. They addressed parental reasons for enrolment, expectations of the program and apprehensions regarding enrolling.

**Results:**

Prior to deciding to enrol, parents tended to be aware of the child’s weight status, had attempted to address it themselves and had sought help from a number of people including health professionals. Parental decision to enrol was influenced by their evaluation of their previous attempts and their child’s emotional state.

**Conclusions:**

Awareness of their child’s weight status is an important first step in parents taking action at this health issue however it is unlikely to be sufficient on its own. Parental decision to join a childhood obesity management program can be complex and is likely to be made after numerous and unsuccessful attempts to address the child’s weight. Strategies to encourage parents to enrol in programs should include activities beyond awareness of weight status.

Health professionals should use contact time with parents to raise awareness of the child’s weight status and to provide encouragement to address overweight and obesity. Parents must be supported in their attempts to address their child’s overweight and obesity whether they choose to manage it themselves or within a program.

## Background

Childhood overweight and obesity has been recognised as a major public health issue [1] and its risks have been well documented [2, 3]. Despite high prevalence of childhood overweight and obesity [1] engaging families in interventions for its management is challenging due to a number of barriers. Whilst some parents may be aware of their child’s weight issue they may not engage in programs due to lack of knowledge of the health consequences associated with overweight and obesity [4], not being concerned about the child’s obesity [4–6] or believing their children will outgrow the excess weight [7]. Some parents who are concerned about their child’s weight status worry that their child will be stigmatised if labelled as obese [8] and fear discussing the weight issue with the child [9].

Another recruitment barrier is lack of parental recognition of their child’s weight issue [4, 5, 9–11] which may be the biggest challenge faced by weight management programs. Parents often underestimate their overweight or obese child’s weight status, appear to be unable to detect a small increase in their child’s weight, and become concerned only when weight gain becomes significant [12]. Further, according to Eckstein et al. [13], parents need to reach a certain level of concern over their child’s weight issue before they take an active approach towards improving it. Whilst it is likely that various factors impact on the level of parental concern which enable parents to enrol in programs for childhood obesity management little is known about these influences. Previous research focused on barriers to recruitment to programs for childhood obesity management rather than on enablers to parental engagement.

Investigating parental reasons for enrolling in programs for childhood obesity management could lead to modifying the programs’ promotion methods, so that recruitment strategies respond more effectively to parents’ needs. Similar issues have been examined with the use of Theory of Planned Behaviour (TPB), in investigations of parents’ perceptions of child feeding [14] and physical activity [15]. The TPB was proposed by Ajzen [16] and suggests that behavioural change is determined by intention to change a behaviour which in turn is influenced by a person’s attitude, subjective norms and perceived control over a behaviour [17]. Application of a TPB in this study could explain how parents progress from being unaware of the child’s weight issue to taking an active approach to address it and enrolling in a program.

While previous research suggested successful recruitment strategies for childhood obesity management programs by investigating barriers to parental engagement [4, 18, 19] there is limited in-depth information about what motivated parents to enrol in such programs. There is a lack of knowledge about parental journey from being unaware to being aware of child’s weight issue, and to developing a concern level which motivates and enables them to enrol into children’s weight management program. Hence, the aim of this study was to explore factors affecting parental decision regarding enrolment into a childhood obesity management program using the TPB.

## Methods

### The sample

In seeking to reduce the prevalence of childhood overweight and obesity an Australian State Health Department funded the delivery of the Parenting, Eating and Activity for Child Health (PEACH™) program to reach 1400 children aged 5 to 11 years who were above the healthy weight range for their age [20]. The PEACH™ program was based on two trials, PEACH [21] and HELPP [22], which demonstrated that a family focused intervention including parenting and healthy lifestyle components is able to achieve sustained weight loss of ~10% in children.

The program is parent-led and involves families attending ten face-to-face facilitated group sessions over a six month period. There was no cost associated with parental participation. The program was delivered in various locations in five stages over 3 years (2013–16); this study was undertaken at the start of stage three of the program delivery.

The study application was reviewed and confirmed as fulfilling the requirements of the National Statement on Ethical Conduct in Human Research by Queensland University of Technology Human Research Ethics Committee (approval number: 1400000413).

### Recruitment

Information about the study was provided via email and telephone to all PEACH™ enrolees by July 2014 (*n* = 154) requesting a reply message should they be interested in participation, which resulted in 13 responses. To enhance recruitment, the research team decided to make follow up telephone calls to the non-responders. Whilst purposeful sampling for diversity in geography, parent and child gender and age of child was attempted, due to time constraints and recruitment taking longer than anticipated follow up telephone calls were made to 51 enrolees who did not respond to the initial email. The research team discontinued follow up telephone calls once 18 enrolees expressed interested in participating in this study. Out of the 31 interested enrolees 21 consented and participated in the study. Their characteristics are reported in Table 1.

**Table 1:** Participant characteristics (*n* = 21)

Characteristics	Number (*n*)
Parent gender	
Male	1
Female	20
Child’s gender	
Male	9
Female	12
Area	
Urban	10
Regional	11
Child’s age (in years)	
5 – 6	4
7 – 8	4
9 – 10	9
11 – <12	4
Sessions completed	
0	2
1–5	12
6–10	7

### Data collection

**Fig. 1:**
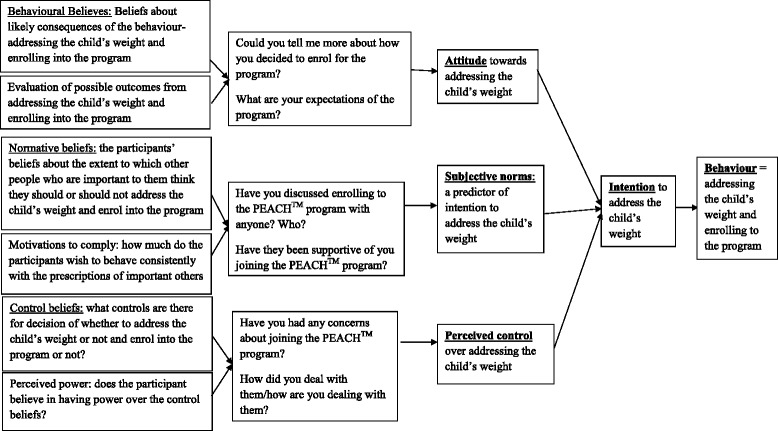
The alignment of the interview questions with constructs of the TPB

The study used a semi-structured qualitative telephone interview with open-ended questions based on the TPB. Figure 1 illustrates the alignment of interview questions with constructs of the theory. The questions were piloted with a convenience sample of two mothers to check face validity, timing of the interview and wording of the questions. Telephone interviewing was employed to increase the opportunity to recruit a diverse sample of participants at various times of the day.

The participants were interviewed over a 5-week period in 2014 by the first author. Hand written notes were taken throughout the interviews. These were expanded upon using the interviewer’s reflection and comments immediately following the interview, transcribed into an electronic file and imported to NVivo software [23].

### Analysis

Prior to analysis, both authors discussed the results and emergent themes. Results were presented to the program’s project and evaluation team for peer review and discussion. All interviews were coded by the first author. Coding difficulties were discussed with the second author to reach a consensus. Data were themed and analysed according to the theoretical constructs of TPB with the use of NVivo software. As the TPB did not appear to fully explain results, the data were further explored by both authors and manually re-coded according to emerging themes and sub-themes.

## Results

### Themes

Three main themes were identified. The first theme, motivation to enrol in the program, gives broader insight into parental journey to joining a program for childhood overweight and obesity management. The other themes, the influence of the support network on program participation and perceived control over children’s weight issue, are related to the TPB constructs namely subjective norms and perceived control respectively. Interview data to support these themes are presented in Tables 2, 3 and 4.

**Table 2:** Theme 1: Comments regarding motivation to enrol in the program

**Sub-theme 1: Parents were aware of children’s weight issue for a long time.**
P7: ‘I have been watching it [weight] for a while, since 2010’
P8: ‘I watched the way he looked for a while’
P10: ‘She’s always been bigger then peers the same age…I’ve been keeping an eye on weight progression and in the last couple of years it increased a lot….she looked chubby’
P12: ‘She’s been overweight since 2–3 years old… she’s always has been at the higher range of overweight and lower range of obese’
P15: ‘It’s always been on the higher end [BMI]’
P16: ‘I wasn’t surprised that he’s overweight because the weight and height has been higher for a while now for age…I got worried when it was flagged by the paediatrician’
P17: ‘She (daughter) is getting older and weight is getting higher and higher’
P18: ‘I was suspected it (child’s overweight) for a while but didn’t want to believe it’
P19: ‘I was concerned for at least 2 years’
P20: ‘I knew for a long time, I was aware since the beginning’
**Sub-theme 2: Visual assessment of children’s weight status (preference for using subjective rather than objective weight assessment methods).**
P1: ‘Weighing [on scales] is not something we do regularly at home…just by looking at her you can see that she is overweight…has a stomach like a pregnant woman’
P8: ‘He’s bigger than average, in comparison to other kids at school…I didn’t put him on scales, I don’t like doing it’
P9: ‘It’s obvious that he’s overweight…just by looking at him…I don’t weigh him but I can see…he’s bigger and taller than others but the body shape is altered…he has man boobs’
P10: ‘I didn’t want to check weight on scales not to emphasise the numbers to him and make a big deal about it’
P12: ‘I never checked weight but you could just see that she’s bigger, especially around the middle’
P13: ‘He’s not proportionally overweight, he doesn’t look overweight…and he is not unfit’
P14: ‘Looks a little chubby’
P15: ‘She is not fat, doesn’t have a big belly like the other kids…when you look at her she doesn’t look overweight’
P16: ‘I noticed that the clothes were getting tighter, she preferred to wear pants with elastic waist rather than jeans…in dancing clothes she was really sucking in her tummy and in comparison to other girls she looks bigger…I never used scales but I noticed that I was struggling to pick her up when giving her a hug’
**Sub-theme 3: Children are aware of their weight issue.**
P4: ‘He notices that he’s bigger than other kids…[he] gets bullied and called fat and lazy’
P6: ‘He realised himself that he is bigger, gets tired easily and asked me why he is slow and fat’
P7: ‘She [daughter] came in crying and said to me [mother] ‘why am I fat…I am fat”
P8: ‘He was aware of his own weight, wanted trendy clothes as the other kids but there was no size for him’
P10: ‘She feels different to other children, told me that she doesn’t want to do sport anymore, gets tired easily, complained that nobody wanted her on their team’
P11: ‘She feels bad about herself and own body…she said ‘I hate myself, I hate my body, just kill me’
P14: ‘He often complains about his red hair, freckles, his tummy, that he is slow and useless. He really brings himself down…he feels that he is just not good enough and weight is just one more things added to it all’
P16: ‘In bath he sometimes tells me: ‘Mummy look at layers on my tummy”
P17: ‘Pretty clothes at the shops don’t fit her…because she has a big tummy…she gets upset because she looks awful’
**Sub-theme 4: Parental concerns about addressing children’s weight and joining PEACH.**
P1: ‘I don’t want her to feel bad about weight’
P4: ‘I spoke to him [about starting PEACH], he was very hesitant…he was worried that it may be another situation when he will be told that he’s fat, eats too much’
P6: 'I was worried what he may think that I think of him, I worried that he may think that I see him as fat'
P8: ‘When I spoke to him [child] about it [starting PEACH] he started crying and said: they will tell me that I’m fat’.
P17: ‘She [daughter] seems to think that skinny is healthy too…media shows only skinny people…children may think that skinny is good, is what the world wants…She [daughter] asked me not to tell anyone at school [about attending PEACH]…she doesn’t want to have attention drawn to her weight’

**Table 3:** Theme 2: Comments about the influence of the support network on program participation

**Sub-theme 1: Participants are not fully supported in their efforts to address the child’s weight**
P2: ‘I mentioned the program to other parents…some discouraged us joining…they thought she will be put on a diet’
P6: ‘I spoke to other parents [about joining the program], they said I shouldn’t enrol because it will make him feel bad about himself’
P8: ‘he [child’s father] didn’t want to come in for the sessions…said ‘why do parents always get the blame?…he’s bad with eating habits too, can’t walk past the fridge without getting something out’
P8: ‘I asked his teacher about the weight but she didn’t think it was an issue’
P12: ‘Some friends said: why would you bother [enrolling to the program]’
P16: ‘I told some parents at school and friends about the program and they said: he’s not fat, don’t put him in a weight loss program’
P20: ‘Friends at work were surprised [to hear about mother joining the program]…they said that she [daughter] is not overweight. I don’t mind telling my side of the family but prefer not to share with my husband side of the family, they are all bigger people and may think: what are you doing it for?’
**Sub-theme 2: Health professionals rarely raise the child’s weight issue with the parents**
P1: ‘I never asked my GP for help with her weight issue…GP never weighs her’
P4: ‘I went to GP to ask for help in the past because we were concerned…GP wasn’t concerned…said that he’s a kid and will grow out of it, things will sort themselves out….he [GP] looked at us and said that he [child] has a bigger frame because we have it too…GP only weighs children when prescribes antibiotics and never commented that there may be an issue with weight’
P5: ‘GP never weighed kids, only when they were younger but no one ever flagged anything. Now kids are past the time when they get weighed by GP anyway’
P12: ‘I was told by GP that things will sort themselves out and that she [daughter] will grow out of it…the only advice given was to eat healthy’
P18: ‘GP was checking weight which was fine but it [weight] wasn’t flagged as an issue’
P19: ‘I have been getting her weight checked at the GP clinic with a nurse…she [nurse] was plotting it all on a graph…didn’t say anything that she [daughter] is overweight but I could see that she is off the graph…I asked her advice [nurse’s], she didn’t have any advice…I asked GP and he said that the main thing is that she [daughter] needs to be active’
**Sub-theme 3: Conversations about the child’s weight issue with health professionals are often considered to be ineffective**
P4: ‘we asked [GP] for referral to a dietitian, it was good…but the dietitian was talking to us…didn’t take the time to talk to him’
P7: ‘We tried so many things…dietitian, GP, paediatrician, no one could help us. I was not impressed with the dietitian, she only spoke to me and didn’t make any effort to build rapport with her [the child]. I thought it was a little waste of time ….I could see that she [the child] is not interested because she was not included in the conversation…and then suddenly was told to eat this or that’
P10: ‘we saw a nutritionist a few times….it was good but a lot [what was recommended] we were already doing at home’
P12: ‘I went to GP and dietitians for help but found that it was not enough, it was food based only…adult focused advice not child focused. They spoke to me more than to her. I regret taking her to the dietitian, she was nice but gave us all these charts and explained it all…she [daughter] at home later on said that she’s so fat according to these graphs and was upset about it’
P13: ‘GP wanted to refer us to dietitian but I declined…I didn’t want him to be worried too much about weight and possibly develop an eating disorder later on’
P15: ‘Once the dietitian took the diet history she said that she’s not sure what we are doing there because we eat very healthy’
P16: ‘We visited a nutritionist at the hospital…it was useful but there was a long wait…the consultation wasn’t long enough and there was no follow up…when we saw a nutritionist later on, it was a different person…it wasn’t very good’
P20: ‘I have been taking him [child] to many doctors, they did many different test but found nothing, at the end they told me: it’s obviously your fault, you let him eat everything and he doesn’t exercise’

**Table 4:** Theme 3: Comments regarding parents’ perceived control over children’s weight issue

**Sub-theme 1: Parents no longer know what to do about children’s weight issue**
P3: ‘I joined it [PEACH] out of shear frustration, I changed my own lifestyle and from just a regular guy became a fitness fanatic…the girls are complete opposite, they are becoming more lazy and fatter every day. They are heading into an opposite direction.’
P8: ‘I’m not sure what to do when he’s hungry all the time…he eats a lot’
P10: ‘It [following healthy lifestyle at home] stopped working, she was getting bigger and her weight was going up quickly…we tried a lot at home but got to a point where it wasn’t enough anymore….I’m willing to try anything’
P13: ‘He’s got problems with eating and acts on impulses….he goes and eats inappropriate foods at night [ice cream at 4 am] when everyone is still asleep’
P17: ‘It’s my fault…we eat healthy on weekdays but eat out on weekends and it’s hard to go back to healthy eating on Monday…I’m worried about holidays…I hope it won’t destroy what they have changed so far’
P20: ‘I was concerned about her weight and attitudes towards food and behaviour. She sometimes steals food from other children’s lunchboxes, complains all the time that she is hungry although there is just no way that it is possible she may be hungry. She is lying about what she had eaten…we didn’t know what to do about it’
**Sub-theme 2: Parents wish for the program to influence their children**
P3: ‘I’m looking for skills to help the girls help themselves, to learn how to teach them skills to help themselves and become healthy’
P4: ‘I joined [PEACH] and wish for him to accept himself, realise that he may not be as slim as other children but he can still be healthy. To understand the feeling of fullness so he doesn’t keep on eating’
P5: ‘I want her to hear about healthy lifestyle from someone else’
P11: ‘I want to give her an opportunity to learn….and understand how food works in the body….to help her with confidence and reassure her that she’s not alone in the word feeling bad’
P12: ‘I was looking for help with educating her, she is old enough to be able to make own decisions about food and may listen better to advice provided by other people than parents’
P13: ‘Kids tend to listen better when it’s [information] coming from someone else’
P17: ‘Sometimes it’s difficult to explain things to kids, what to eat, what to do, so they sometimes listen better when these things come from other people’
**Sub-theme 3: Non-parental influences on children’s weight status**
P1: ‘He [ex-partner and child’s father] must learn more about nutrition because he feeds the kids pasta and potatoes five times a week’
P3: ‘I’m trying to role model but find that management of it all (weight issue) is hard because their mother [ex-partner] does not support my efforts’
P12: ‘Media definitely plays a role here…programs like the Biggest Loser show kids that it’s ok to pick on fat people… Advertising makes it very hard for families with a child who is overweight…kids play sport and only junk food is in cafeteria and junk food vouchers are given as rewards [after games]. Dealing with fast food ads and children and healthy eating is very hard’
P14: ‘Media definitely influences children’s food choices, they want yoghurts with their favourite characters, meals with toys…when eating out none of the kids’ meals options at restaurants are nutritious…we buy them an adult meal because is it more nutritious’
P15: ‘Media shows unrealistic images of very thin children. No one looks like that’
P18: ‘Media plays a huge role…advertising food, bad food…kids are drawn to it and then want to get it. It’s difficult to deal with it.’
P19: ‘I’m annoyed that in children’s shows you can see healthy weight range children eating crap food and no one talks then about the food being healthy or not…not everyone has a weight problem but there should be something said there about it.’
P20: ‘Media keeps on advertising fast food, and you get toys. They try to say they have healthy options but have you ever hear a child saying – mum can I have yoghurt when at Maccas? They all want the happy meal.’

### Theme 1: Motivation to enrol in the program (Table 2)

Parental motivation to enrol in the program appeared to be influenced by two main factors. First, parental awareness of their child’s weight issue and recognition of the severity of the problem, and second, children’s self-awareness of their overweight and obesity.

The participants were aware of their children’s weight issue long before enrolling to the program and reported ‘watching’ the weight status as the child grew older. Two methods of assessing children’s weight status were used; objective (weighing children on scales and/or getting their measurements taken and assessed by health professionals) and subjective (eyeballing the child’s body size, observing how their clothes fit and comparing their body size to other children). While the majority of participants reported using both of these methods, it seemed that their judgement regarding the severity of the weight issue was mostly based on subjective methods.

Undertaking an objective assessment of children’s weight status was seen as useful in tracking children’s growth however only few used this approach. A few participants reported being aware of the children growth charts but did not base their perception of their child’s weight status using this tool; instead the participants appeared to rely on a subjective assessment of their child’s weight status. When using the subjective methods, most commonly the participants undertook visual assessment (eyeballed) of their child’s weight status and emphasised their child having a large stomach. Some participants reflected on their child’s body size with regard to clothes they had to purchase for them (being in an adolescent or adult size at an early age) or not being able to get fashionable items wanted by their child due to poor fit or lack of size. A number of participants commented on their child’s weight status by making comparisons with other children’s body shape. This often allowed some participants to emphasise the severity of their child’s weight issue, when they saw smaller children in comparison to their own child, and allowed others to point out that their child’s weight issue is not at extreme level, when comparisons were made to larger children.

Parental motivation to enrol in the program was also influenced by their child’s self-awareness of their overweight and obesity. Some participants commented on their children being aware and sometimes upset about their body size. Several children were reportedly bullied and teased about their weight and did not participate in sport activities, either by own choice or due to being excluded by other children. Some participants also reflected that their children noticed they are ‘bigger and slower’ than others and cannot buy or fit into the same clothing as their peers. Participants recalled being asked by their children about the reason for their weight issue; and some children reportedly cried and put themselves down due to their weight status. When the participants spoke to their children about joining the program, the children were often hesitant and concerned about how they will be treated in the program, and sometimes needed convincing to attend.

### Theme 2: The influence of the support network on the program participation (Table 3)

The participants sought support and/or advice with regard to their children’s weight issue from one or more persons (family member, health professional, another parent and/or teacher). While most of the participants’ family members and partners appeared to be supportive, a few of them failed to recognise the child’s weight issue and/or own responsibility to address it. Most of the participants who were separated from the child’s other parent faced challenges in engaging the other parent in identification as well as management of their child’s weight issue. Some participants turned for support to their friends and colleagues who appeared to be surprised to hear they wanted to address the child’s weight and discouraged enrolling in the program.

In addition, several participants consulted with health professionals (general practitioners (GPs), paediatricians, nutritionists and/or dietitians) their children’s weight issue and/or enrolling into the program. Those who spoke to their GP initiated the conversation themselves and while a few participants received support others remembered being reassured that their child will outgrow of being overweight and that ‘things will sort themselves out’. Consultations with dietitians and nutritionists were rare; however, those who attended them were usually unimpressed with the service delivery and/or unsatisfied with the information given. The advice provided by GPs, paediatricians or dietitians was spoken of as ‘adult-focused’, ‘food-based’ and generally ‘not enough’.

### Theme 3: Perceived control over children’s weight issue (Table 4)

The majority of participants appeared to reach a breaking point in managing their child’s weight where, despite following healthy lifestyles and role modelling, nothing worked and children continued to put the weight on. One participant described coming to a point where they were ‘willing to try anything’. A few participants expressed concerns regarding their children’s behaviours around food and eating; one child was reportedly eating during the night and another child was stealing food from others and lying about it to his/her parents. The participants thought that their children may be more receptive to healthy lifestyles messages coming from someone else other than the parents, and that some children were old enough to make own choices. Hence, they joined the program in a hope that it will influence their child’s eating behaviours, physical activity and self-esteem and that the child will learn how to make healthy decisions in life. This is despite the program being promoted as focusing on parents as agents of change.

Children’s weight issue was often spoken of as a problem beyond parental control due to influences of other people and the environment. These included unhealthy feeding practices of the child’s other parent, media, and advertising as well as food choices in cafeterias and restaurants. Some participants expressed their frustration and annoyance with television programs portraying children eating unhealthy food with no mention of the food quality. Other participants reported finding it hard to deal with children’s weight issue when food companies use toys and children’s favourite characters to attract them to unhealthy items. Whilst only two participants expressed feeling responsible for their child’s weight issue, all agreed being uncertain of what else can be done to manage their child’s weight and eating issues, which led them to joining the program.

## Discussion

Findings of this study suggest that there are many factors influencing parents’ decision to enrol into programs for childhood overweight and obesity management. Parents tended to be aware of their child’s weight issue prior to enrolment, they had tried to manage it themselves prior to joining the program and sought help and support from health professionals, family and friends. The factors which influenced the decision to enrol in the program, once parents are aware of the child’s weight issue, appeared to be related to parental evaluation of their attempts to manage the problem, the options for obesity management that are available, the influences on their decision of what to do next as well as their perceived control over addressing the weight issue.

The study design was based on TPB however this framework failed to fully explain the results. The TPB was applied in this study to get insights into what enables parental shift from intention to change health behaviour to the actual behaviour change (addressing child’s weight status). However, the results indicate the participants have previously attempted to manage their child’s weight status which highlights that the transition from intention to behaviour change occurred long before the participants enrolled in the program. This emphasises that participants’ engagement with the childhood obesity management program was not their first attempt to address their child’s weight issue. They decided to enrol in the program whilst being influenced by factors beyond those explained by TPB, such as their perception of the severity of child’s weight issue and child self-awareness of their body size, which is why further data analysis was undertaken. Overall, programs for childhood obesity management should consider barriers and enablers which affect parental decision to enrol to design effective recruitment strategies and enhance participation.

The participants had been aware of their child’s overweight or obesity however the majority commented on severity of their child’s weight issue by making body size comparisons to other children. Similar to previous research, this is supported by the social comparison theory [24] which suggests that people judge themselves by making comparisons with others rather than against an absolute scale [25]. In some cases this approach may act as a motivator for action, when parents use upward comparison and their child appears to be bigger than others. However in other cases, when parents use downward comparison and their child is visually smaller than others this may result in absence of action. As previous evidence suggests that parents are unable to accurately perceive their children’s body weight [24, 26–29], especially with a rise in prevalence of overweight and obesity [30], by trusting subjective assessment methods parents may falsely believe that their weight management strategies are sufficient resulting in lack of engagement in programs such as PEACH™.

The results of this study identify opportunities for assisting families in making a decision to address their child’s weight and join a program such as PEACH™. As in a previous study [31], most commonly, participants asked their GPs for advice with managing their children’s weight. It is concerning that many were discouraged from taking an active approach in managing their children’s weight despite research suggesting that primary care physicians’ (including GPs) are mostly aware that children do not outgrow from being overweight [32]. Overweight and obese children are likely to continue on this trajectory into adulthood [33–35] which has both health and economic consequences [36]. Further, the fact that the majority of participants who sought help from their GPs reported having to initiate the conversation themselves indicates that health professionals do not routinely weigh children, interpret and communicate results to the parents [12, 37, 38]. Research suggests that while GPs fail to weigh children and anticipate a negative parental response to weighing their child, parents mostly find such practice useful and majority feel either neutral or positive about it [39]. It appears that despite having opportunities to discuss children’s weight issue and its management with parents, health professionals may not only fail to flag the weight problem but they also may halt parental efforts to address the issue when ideally they could support and encourage it.

The findings of this study suggest that parents who engage in programs for childhood overweight and obesity management may have been made aware of their child’s weight issue long before expressing interest in the program. Moreover, enrolled parents may have attempted to manage their child’s overweight or obesity prior to starting a program for its management. The results signify that parental engagement in a program may not be an equivalent to starting taking action towards addressing their child’s overweight and obesity because many of this study’s participants tried managing their child’s weight issue prior to enrolment. Hence, programs should acknowledge that parental recognition of the issue as well as attempted behavioural change may have occurred long before commencing the program, and recruitment strategies may need to be modified to respond to parents’ needs. Furthermore, appropriate services should be available to parents at any stage of their journey of addressing their child’s weight issue; to parents who choose to manage their child’s overweight and obesity on their own as well as those who seek support from a program.

Parents’ decision to join childhood overweight and obesity management program may not be the first reaction to gaining knowledge of child’s weight issue. In this study, many parents who were aware of their child’s weight issue attempted controlling it on their own and decided to engage in PEACH™ after realising their efforts are unsuccessful. Hence, gaining awareness of this issue may not necessarily influence parental decision to join programs such as PEACH™ when these are available in the community. In agreement with Eckstein et al. [13] in addition to gaining awareness of child’s weight issue there is a need for development of a certain degree of concern before any action occurs. Parents, who are aware of the child’s overweight or obesity and see their current efforts as sufficient in managing the problem, even if in reality these are ineffective, may not seek support from a program. Although there may be some level of concern about child’s weight it may not have reached a point where it motivates parents to enrol in a program. In the case of this study, the level of concern which led to parental engagement appeared to be when parents felt hopeless in what else to do about child’s weight issue, which in some cases was a result of the child’s sadness, frustration and dissatisfaction from their body weight. This poses a challenge for childhood overweight and obesity management programs, as to how to attract parents who may be attempting to address their child’s weight issue prior to them reaching point of despair. Further, health services should acknowledge that some parents may choose to manage their child’s overweight and obesity on their own, and provide sufficient support for these parents throughout their journey to ensure it is successful.

### Limitations

These findings should be considered in the context of a few limitations. This study’s small sample size limited its ability to thoroughly investigate differences between participants’ responses and their gender, geographical location or children’s age. This study population was families with overweight and obese children enrolled in a management program hence the perspectives of this convenience sample may not be representative of other parents with overweight and obese children nor those enrolled in management programs. Further, the program’s enrolment process only collected limited demographic data therefore information on parental socioeconomic or marital status was not available for this study. This study did not analyse perspectives of parents according to their child’s weight status (overweight versus obese). Parental reasons for enrolment in programs for childhood overweight and obesity management may vary according to severity of their child’s obesity hence further research studies with larger sample sizes should consider distinguishing between data from parents of overweight and obese children. Furthermore, the interviews were not audio-recorded, which may have resulted in failure to record some information and may have introduced information bias into this study. While the authors discussed the results prior to the first author coding data independent coding by the second author would have increased the interrater reliability.

## Conclusion

This study adds to the evidence exploring factors influencing parental decision to address children’s weight issue and enrol to programs for childhood overweight and obesity management. Parental decision to join a childhood obesity management program can be complex and is likely to be made after numerous and unsuccessful attempts to address the child’s weight. These findings provide an insight into what parents who enrol into a childhood weight management program go through prior to recruitment. There are various ways in which management of child’s overweight and obesity can be understood and enrolling into a program which addresses this issue is only one of them. Making parents aware of their child’s overweight and obesity may not result in an increased interest in childhood weight management programs but may move parents closer to taking action toward addressing the issue. Parents must be supported in their attempts to address their child’s overweight and obesity whether they choose to manage this issue themselves or within a program. The challenge is to provide sufficient and adequate health services and programs to engage parents of overweight and obese children in active management early and before they reach a point of despair.

## Abbreviations

GP: General practitioner
HELPP: Healthy eating, local policies and programs
PEACH: Parenting, eating, activity for child health
TPB: Theory of planned behaviour
